# Optimizing Movement for Maximizing Lifetime of Mobile Sensors for Covering Targets on a Line

**DOI:** 10.3390/s19020273

**Published:** 2019-01-11

**Authors:** Peihuang Huang, Wenxing Zhu, Longkun Guo

**Affiliations:** 1 College of Physics and Information Engineering, Fuzhou University, Fuzhou 350116, China; peihuang.huang@foxmail.com; 2 College of Mathematics and Computer Science, Fuzhou University, Fuzhou 350116, China; wxzhu@fzu.edu.cn

**Keywords:** mobile sensor, 𝒩𝒫-hard, target coverage, line boundary, optimal solution

## Abstract

Given a set of sensors distributed on the plane and a set of Point of Interests (POIs) on a line segment, a primary task of the mobile wireless sensor network is to schedule covering the POIs by the sensors, such that each POI is monitored by at least one sensor. For balancing the energy consumption, we study the min-max line barrier target coverage (LBTC) problem which aims to minimize the maximum movement of the sensors from their original positions to their final positions at which the coverage is composed. We first proved that when the radius of the sensors are non-uniform integers, even 1-dimensional LBTC (1D-LBTC), a special case of LBTC in which the sensors are distributed on the line segment instead of the plane, is NP-hard. The hardness result is interesting, since the continuous version of LBTC to cover a given line segment instead of the POIs is known polynomial solvable. Then we present an exact algorithm for LBTC with uniform radius and sensors distributed on the plane, via solving the decision version of LBTC. We argue that our algorithm runs in time $O(n^{2}\log n)$ and produces an optimal solution to LBTC. The time complexity compares favorably to the state-of-art runtime $O(n^{3}\log n)$ of the continuous version which aims to cover a line barrier instead of the targets. Last but not the least, we carry out numerical experiments to evaluate the practical performance of the algorithms, which demonstrates a practical runtime gain comparing with an optimal algorithm based on integer linear programming.

## 1. Introduction

In the past decades, wireless sensor networks have brought tremendous changes to human society and proposed many technique challenges. Among them, the coverage topics including area coverage [1] and barrier coverage [2] are among the hop spots that attract lots of research interest. In area coverage, the task is to schedule the new positions of the sensors, such that each point in the given target region is covered by at least one sensor. Differently, in barrier cover the task is to monitor only the boundary of a given region, and the aim is to guarantee that intruders can be detected when they are crossing the barrier. Comparing to area coverage, barrier coverage has an advantage of using significantly less sensors and hence is scalable for large scale wireless sensor networks (WSN). Furthermore, some applications only require a set of Points Of Interest (POIs) along the boundary to be monitored. In the context, a problem arises how to guarantee every POI on the barrier to be covered. The current-state-of-art method is to first cover POIs using the stationary sensors, and then use mobile sensors to cover every not-yet covered POI along the barrier. For the second phase, we traditionally have the following assumptions for the modeling: (1) Sensors are acquired with mobile ability; (2) The initial positions of the sensors are distributed on the plane, and the POIs are distributed along a line segment (Although the shape of the boundary can be various, most researches nonetheless focus on line boundary because curves in other shapes can be considered as a variable of line segments); (3) The aim of sensor networks is to prolong the lifetime. This arises the min-max 2D Line Boundary Target Coverage problem (min-max 2D-LBTC) as follows:

**Definition** **1.** *Let P and* Γ *be respectively a set of POIs distributed in a line segment [0,L] and a set of mobile sensors distributed on the plane, where j∈P has a position $\left(p_{j},\;0\right)$ while i∈Γ has a position $\left(x_{i},\;y_{i}\right)$ and a positive sensing radius $r_{i}$. The min-max* 2D-LBTC *problem aims to compute a new position $\left(x_{i}^{\prime },\;0\right)$ for each sensor i∈Γ, such that each POI j∈P is* covered *by at least one sensor, and the maximum movement of the sensors from their original positions to the new positions is minimized that $\max_{i\in \Gamma }\left\{\left.\sqrt{{\left(x_{i}-x_{i}^{\prime }\right)}^{2}+{y_{i}}^{2}}\right|i\in \Gamma \right\}$ is attained, where j∈P is* covered *means there exists a sensor i∈Γ with position $\left(x_{i}^{\prime },\;0\right)$ that $x_{i}^{\prime }-r_{i}\leq p_{j}\leq x_{i}^{\prime }+r_{i}$.*

When no confusion arises, we shall use LBTC short for the min-max 2D-LBTC problem for briefness. In particular, we use one dimensional min-max Line Boundary Target Coverage problem (1D-LBTC) to denote the special case of LBTC when the initial positions of all the sensors are also distributed on the line boundary. Moreover, the decision version of LBTC (decision LBTC for short) is, for a given movement bound *D*, to determine whether there exists a feasible coverage with each sensor’s movement bounded by *D*. Besides, when the aim is to cover the line boundary itself instead of the POIs thereon, we respectively have the min-max Line Boundary Coverage (LBC) problem and one-dimensional-LBC (1D-LBC) problem, which have already been well studied and a number of algorithms have been developed. All the notations of this paper are summarized as in Table 1.

### 1.1. Related Works

To the best of our knowledge, Kumar et al. [2] were the first to consider the boundary (barrier) coverage problem using sensors against a closed curve (i.e., a moat), via transforming the coverage problem to the path problem of determining whether there exists a path between two specified nodes, although the research of barrier coverage started from early 90s due to Gage [3]. The algorithm from Kumar et al. is scalable and can also be extended to solve the *k*-coverage problem by transforming to the *k*-disjoint path problem. However, the disadvantage is that it can only be used to determine whether a coverage exists using the deployed *stationary* sensors. A problem for stationary sensors is that, after deployment there might exist no coverage over all POIs. For the case, a state-of-art solution is to employ mobile sensors to fill the gaps between the stationary sensors. In the scenario, the WSN applications would require to maximize the minimum lifetime of the mobile sensors or to minimize the total energy consumption. For the former, the aim is to schedule new positions for the mobile sensors such that the barrier is completely covered, and that the maximum movement of the sensors is minimized as to prolong the lifetime of the WSN. When the sensors are on the line of the barrier, the 1D-LBC problem is shown optimally solvable in $O(n^{2})$ time for uniform radii in Paper [4]. The same paper has also proposed an algorithm with O(n) time for uniform radii and $\sum_{i}r_{i}\leq L$, and with $x_{1}\leq \cdots \leq x_{n}$ for the sensor $\Gamma =\{s_{1},\;\cdots ,\;s_{n}\}$, where *L* is the length of the barrier, *n* is the number of the sensors. Later, Chen et al have improved the time complexity to O(nlogn) for uniform sensor radii and proposed an $O(n^{2}\log n)$ time algorithm for non-uniform radii in paper [5]. Besides straight line barrier, circle/simple polygon barriers has been studied and two algorithms have been given developed by Bhattacharya et al. in [6], which have an $O(n^{3.5}\log n)$ time relative to cycle barriers and an $O(mn^{3.5}\log n)$ time relative to polygon barriers, in which *m* is the number of the edges on the polygon. The later time complexity was then decreased to $O(n^{2.5}\log n)$ in [7]. For the more generalized case in which the sensors are distributed on the plane, the LBC problem is known to be strongly NP-hard for sensors with general integer sensing radius [8], while LBC using uniform radius sensors is shown solvable in $O(n^{3}\log n)$ time [9]. Although these elegant algorithms have been developed for LBC for both 1D and 2D setting, none of them is applicable to LBTC since as we shall show in the paper, LBTC is NP-complete for non-uniform radius.

Other than the Min-Max case, there are also applications require min-sum coverage that is to minimize the total energy consumption, which is to minimize the total movement of the mobile sensors. For this objective, Min-Sum LBC, which aims to minimize the sum of the movements of all the sensors, were studied in literature. Min-Sum LBC was shown NP-complete for arbitrary radii while solvable in time $O(n^{2})$ for uniform radii by Czyzowicz et al. [10]. The Min-Num relocation problem of minimizing the number of sensors moved, is also proven NP-complete for arbitrary radii and polynomial solvable for uniform radii by Mehrandish et al. [11]. A PTAS has been developed for the Min-Sum relocation problem against circle/simple polygon barriers by Bhattacharya et al. [6], which was later improved to an $O(n^{4})$ time exact algorithm by Tan and Wu [7]. For covering a barrier with Min-Sum movement, the most recent result is a factor-$\sqrt{2}$ approximation algorithm for covering POIs along a barrier using uniform-radius sensors, aiming to minimize the sum of the movement [12]. However, it remains open whether the min-sum LBC problem is NP-hard. For target coverage task in the plane, Liao et al. have develop algorithms for Min-Sum movement to minimize the total consumed energy [13].

### 1.2. Our Results

In this paper, we first show that 1D-LBTC is NP-hard when the sensors are with non-uniform integer radii by proposing a reduction from the 3-partition problem that is known strongly NP-complete [14]. This  hardness result is interesting, because 1D-LBC, the continuous version of 1D-LBTC, is shown solvable in polynomial time $O(n^{2}\log n)$ [5]. This means LBTC and LBC belong to different classes of computational complexity.

Then, we propose a sufficient and necessary condition to determine whether there exists a feasible cover for the barrier under the relocation distance bound *D*. Based on the condition, we propose a simple greedy approach that outputs “infeasible” if $D<D^{*}$, and otherwise computes a feasible solution under the movement bound *D*, such that the sensors cover all the POIs in their new positions. We show that the decision algorithm is with a runtime O(nlogn). By employing the binary search technique, we propose an algorithm using the decision algorithm as a routine to actually find a minimum integer movement bound $D=D^{*}$, when $D^{*}$ is integral. The algorithm takes $O(n\log n\log d_{max})$ time, where $d_{max}$ is the maximum distance between the sensors and the POIs.

For instances with $D^{*}$ being a real number or with large $d_{max}$, we propose another algorithm that employs the binary search method against $O(n^{2})$ possible values of $D^{*}$ instead of the continuous value range. The trick is to construct the set of all possible values of $D^{*}$ and show its size is $O(n^{2})$. This improves the runtime of the algorithm to $O(n^{2}\log n)$, which is the time needed to sort the $O(n^{2})$ possible values of $D^{*}$. The later algorithm remains correct even when *D* is allowed to be any real number. In contrast, the former algorithm can only work for integer $D^{*}$. To the best of our knowledge, our algorithms are the first polynomial algorithms for LBTC.

The following paragraphs will be organized as follows: we shall first give the NP-completeness proof in Section 2; Then present the algorithm for Decision LBTC with uniform sensor radii together with the correctness proof in Section 3; Next, actually solve the LBTC problem by employing the binary search method first against a continuous range, and then over our proposed discrete set in Section 4; After that, evaluate the proposed algorithms via experiments in Section 5; At last, conclude the paper in Section 6.

## 2. NP-Completeness of Decision 1D-LBTC

In this section, we shall show the decision LBTC problem is NP-complete when the sensors are with non-uniform integer radii, by giving a reduction from the 3-partition problem that is known strongly NP-complete [14]. In 3-partition, we are given a set of 3n integers $U=\{a_{1},\;\cdots ,\;a_{3n}\}$ with $\sum_{i=1}^{3n}a_{i}=Bn$ for an integer B>0. The aim is to determine whether U can be divided into *n* subsets, such that each subset is with an equal sum *B*.

**Theorem** **1.** *Decision 1D-LBTC is NP-complete when the sensors are with non-uniform integer radii.*

The key idea of the construction of a reduction from 3-Partition to the decision LBTC problem is to model $a_{i}\in U$ as the diameter of the sensors, and place POIs on the line segment in a way that a coverage of the POIs is actually a partition of the numbers in U. More detailed, for a given instance of 3-Partition, the construction of the corresponding instance of decision LBTC is simply as below:

1. Construct a line segment with length (2n−1)B;
2. Place 4nB POIs on the line barrier as below:
  (a) Decompose the line segment into 2n−1 subsegments with equal length;
  (b) Select *n* sections from the subsegments, where the *i*th section is the (2i−1)th subsegment;
  (c) **For** the *i*th *section*, i=1,⋯,n, **do**
      **For** j=0,⋯,B−1 **do**
           Put two POIs respectively to the two positions (2(i−1)B+j+jϵ,0) and (2(i−1)B+j+1−(B−j)ϵ,0), where ϵ is a small positive number;
      **End for**
    **End for**
3. Place 3n sensors on position (0,0), where sensor *i* is with radii $\frac{a_{i}}{2}$;
4. The maximum movement is set as D:=(2n−1)B.

**Figure 1:**
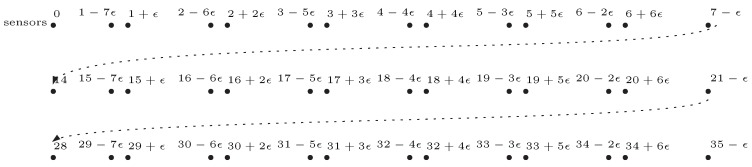
An example of the construction of 1D-LBTC against a 3-Partition instance U={1,1,2,2,2,3,3,3,4,}, n=3 and B=7. There are two sensors with diameter 1, one with diameter 4, three with diameter 2 and the other three with diameter 3, whose original positions are all on (0,0). The movement bound is set D=35.

An example of the above construction is as depicted in Figure 1. Note that, the instance of decision 1D-LBTC constructed above contains 2nB POIs and 3n sensors. Anyhow, 3-Partition is known strongly NP-complete, which means, 3-Partition remains NP-complete even when *B* is polynomial to *n*. Therefore, the construction can be done in polynomial time for *B* being polynomial to *n*.

The main idea behind the construction is to construct a relationship between the number of covered POIs and the diameters of the sensors that are actually the integers in U. More precisely, the property on the relationship is as in the following:

**Proposition** **1.** *Against a 1D-LBTC instance produced by the above construction, a sensor with diameter 2r can cover at most 4r POIs.*

**Proof.**  When a sensor is with a diameter 2, apparently it can cover at most 4 POIs. Suppose the proposition is true for sensors with diameter smaller than 2r. Then, let $r_{1}+r_{2}=r$ be two positive integers smaller than *r*. By induction, we have that sensors with diameters $2r_{1}$ and $2r_{2}$ can cover upto $4r_{1}$ and $4r_{2}$ POIs, respectively. In addition, the two sensors with radii $r_{1}$ and $r_{2}$ can cover as many POIs as a sensor with a radii $r=r_{1}+r_{2}$ does. Therefore, the sensor with diameter 2r can cover no more than $4r_{1}+4r_{2}=4r$ POIs. This completes the proof. □

**Lemma** **1.** *An instance of 3-Partition is feasible if and only if the corresponding 1D-LBTC instance is feasible.*

**Proof.**  Suppose the instance of 3-Partition is feasible. Without loss of generality, we assume that $\left\{U_{i}|i=0,\;\cdots ,\;n-1\right\}$ is a solution to the 3-Partition instance which divides U to a collection of *n* sets, among which $U_{i}=\left\{a_{l_{i}+1},\;\cdots ,\;a_{l_{i+1}}\right\}$ and $l_{0}=0$. Since D=(2n−1)B equals the length of the barrier and the original position of each sensor is (0,0), each sensor can be moved any point of the barrier. Then we need only to use the sensors in $U_{i}$, which are with radius $a_{i_{j}},\;\cdots ,\;a_{i_{j+1}}$ and with a sum exactly *B*, to cover the segment from 2iB to (2i+1)B. That apparently results in a coverage for all the POIs in the *i*th section.

Conversely, suppose the corresponding LBTC instance is feasible. Then since sensor *j* with radii $\frac{a_{j}}{2}$ can at most cover $2a_{j}$ continuous POIs, and each section contains exactly 2B POIs, so the diameter sum of the sensors for each section is at least *B*. Then because the diameter sum of all the sensors is Bn, and there are *n* sections, the diameter sum of the sensors for each section is exactly *B*. Therefore, the diameters for the sensors for the sections is a solution to the corresponding instance of 3-Partition. □

From the fact that 3-Partition is strongly NP-complete, and following a similar idea of the above proof for Theorem 1, we immediately have the following hardness for LBTC:

**Corollary** **1.** *Decision 1D-LBTC is strongly NP-complete.*

## 3. A Greedy Algorithm for 2D-LBTC with Uniform Sensors

The basic idea of the algorithm is to cover the POI from left to right, preferably using sensors that are likely less useful for later coverage. More precisely, let $\left[l_{i},\;g_{i}\right]$ be the possible coverage range of sensor *i*, where $l_{i}$ and $g_{i}$ are respectively the positions of the leftmost and the rightmost POIs, with respect to the given distance *D*. That is, $l_{i}$ and $g_{i}$ are the leftmost and the rightmost positions of POIs sensor *i* can cover within movement *D*. Then the key idea of our algorithm is to cover the POIs from left to right, using the sensor that can cover the leftmost uncovered POI within movement *D* and is with minimum $g_{i}$.

The algorithm is first to compute its possible coverage range $\left[l_{i},\;g_{i}\right]$ for each sensor *i* with respect to the movement constraint *D*. Apparently, $\left(x_{i},\;0\right)$ is the projective point of sensor *i* on the line, so we have $l_{i}=x_{i}-\sqrt{D^{2}-y_{i}^{2}}$ and $g_{i}$=$x_{i}+\sqrt{D^{2}-y_{i}^{2}}$ for each sensor *i*. Then, the algorithm starts from point s=(0,0), to cover the line from left to right. The algorithm prefers using the sensor with a small $g_{i}$, since a sensor with a large $g_{i}$ would has a better potential to cover the POIs on the right part of the line.

Let *s* be the position the uncovered leftmost POI on the line barrier. Then among the set of sensors $\left\{i|l_{i}\leq s\leq g_{i}\right\}$, the algorithm repeats selecting the sensor with minimum $g_{i}$ to cover the uncovered POIs of the line barrier starting at *s*. Note that $\left\{i|l_{i}\leq s\leq g_{i}\right\}$ is exactly the set of sensors that can monitor a set of uncovered POIs starting at *s* by relocating at most *D* distance. The algorithm terminates either the set of POIs are completely covered, or the instance is found infeasible (i.e., there exists no unused sensor *i* with $l_{i}\leq s\leq g_{i}$ while the coverage is not yet done). The algorithm is formally as in Algorithm 1.

Note that Algorithm 1 takes O(n) time to compute $l_{i}$ and $g_{i}$ for all the sensors in Steps 2–3, and takes O(nlogn) time to assign the sensors to cover the targets on the line barrier in Steps 4–15. Therefore, we have the time complexity of the algorithm:

**Lemma** **2.** *Algorithm 1 runs in time $O\left(n\log n\right)$.*

Before proving the correctness of Algorithm 1, we need the following lemma stating the existence of a special coverage for a feasible LBTC instance.

**Proposition** **2.** *Let $\left(x_{j},\;y_{j}\right)$ be the position of sensor j in the plane. Assume $p_{1}\left(s,\;0\right)$, $p_{2}\left(x_{j}^{\prime },\;0\right)$ and $p_{3}\left(x_{j}^{\prime \prime },\;0\right)$ are three points on a line segment. If $s\leq x_{j}^{\prime \prime }\leq x_{j}^{\prime }$, then $d\left(j,\;p_{3}\right)\leq \max\left\{d\left(j,\;p_{1}\right),\;d\left(j,\;p_{2}\right)\right\}$ holds. That is, the distance between the sensor and the middle point is not larger than the larger distance between the sensor and the other two points.*

**Lemma** **3.** *If an instance of LBTC is feasible, then there must exist a coverage in which the sensors are s-ordered.*

**Algorithm 1** A greedy algorithm for decision LBTC**Input:** A bound $D\in Z^{+}$ on maximum movement, a set of sensors Γ={1,⋯,n} with original
      positions $\left\{\left(x_{i},y_{i}\right)|i\in {\left[n\right]}^{+}\right\}$ and *r* being the sensing radii, a set of POIs P={1,⋯,m} with      positions $p_{1}⪯p_{2}⪯\cdots ⪯p_{m}$, where $p_{j}$ is the position for j∈P;**Output:** New positions $\left\{x_{i}^{\prime }|i\in {\left[n\right]}^{+}\right\}$ for the sensors or return “infeasible”. 1: Set I:=Γ and $s:=p_{1}$, where *s* is the leftmost point of the uncovered part of the barrier. 2: **For** each sensor *i*
**do** 3:    Compute the leftmost position $l_{i}$ and the rightmost position $g_{i}$ that sensor *i* can monitor; 4: **EndFor** 5: **While**
I≠∅
**do** 6:    **If** there exists $i^{\prime }\in I$, such that $l_{i^{\prime }}\leq s\leq g_{i^{\prime }}$
**then** 7:       Select the sensor with minimum $g_{i}$ among all the sensors $\left\{i^{\prime }|l_{i^{\prime }}\leq s\leq g_{i^{\prime }}\right\}$, which is to find            sensor i∈I that $g_{i}=\min_{i^{\prime }:\;l_{i^{\prime }}\leq s\leq g_{i^{\prime }}}\left\{g_{i^{\prime }}\right\}$; 8:       Set $t:=\min\{s+2r,\;g_{i}\}$ and $x_{i}^{\prime }:=t-r$; 9:       Set $s:=\min\{p_{j}|p_{j}>t\}$ and I:=I∖{i};10:    **Else**11:       Return “infeasible”;12:    **End if**13:    **If**
$t\geq p_{m}$
**then** /*All POIs have been covered. */14:       Return “feasible” together with the new positions $\left\{x_{i}^{\prime }|i\in \Gamma \right\}$;15:    **Endif**16: **Endwhile**

**Proof.**  The key idea of the proof is that, any coverage of LBTC that is not *s*-ordered, can be converted to an *s*-ordered coverage by re-scheduling the sensors of covering the POIs.

Suppose there exist two sensors *i* and *j*, such that $g_{i}>g_{j}$ but $x_{i}^{\prime }<x_{j}^{\prime }$. Then we need only to swap the final positions of *i* and *j*, i.e., to simply set the new final positions $x_{i}^{\prime \prime }$ and $x_{j}^{\prime \prime }$ of sensor *i* and *j* as below:

**If** $x_{i}^{\prime }-r\geq s$ **then**

   Set $x_{i}^{\prime \prime }:=x_{j}^{\prime }$ and then $x_{j}^{\prime \prime }:=x_{i}^{\prime }$;

**Else**

   Set $x_{i}^{\prime \prime }:=x_{j}^{\prime }$ and $x_{j}^{\prime \prime }=s+r$.

**EndIf**

Apparently, the POIs exclusively covered by *i* are now covered by sensor *j*, and *vice versa*. So after the swap the sensors will remains a coverage for the POIs on the line. It remains to show the swap will not increase the maximum movement. Recall that the leftmost and the rightmost points sensor *j* can cover are respectively $l_{j}$ and $g_{j}$. Because sensor *j* can move to $x_{j}^{\prime }$ under the movement bound *D*, we have

(1) $$l_{j}\leq x_{j}^{\prime }-r\leq x_{j}^{\prime }+r\leq g_{j}\leq g_{i}.$$

On the other hand, in either case of the swap, we have $x_{i}^{\prime \prime }=x_{j}^{\prime }\geq x_{i}^{\prime }$. So combining Inequality (1), we have $l_{i}\leq x_{i}^{\prime \prime }-r\leq x_{i}^{\prime \prime }+r\leq g_{i}.$ That means

$$l_{i}+r\leq x_{i}^{\prime \prime }\leq g_{i}-r.$$

Then following Proposition 2, the distance between sensor *i* and its new position $x_{i}^{\prime \prime }$ is bounded by $D=\max\{d\left(i,\;\left(l_{i}+r,\;0\right)\right),\;d\left(i,\;\left(g_{i}-r,\;0\right)\right)\}$. The case for the new position of sensor *j* is similar except that the distance between sensor *j* and its new position $x_{i}^{\prime \prime }$ is bounded by $D=\max\{d\left(j,\;\left(\max\left\{s,\;l_{j}+r\right\},\;0\right)\right),\;d\left(i,\;\left(g_{i}-r,\;0\right)\right)\}$. This completes the proof. □

Based on Lemma 3, given a feasible instance of LBTC, we can assume there exists an *s*-ordered coverage, say $\Gamma^{\prime }=\left\{s_{1},\;\cdots ,\;s_{k}\right\}$ which is the set of sensors used to compose the coverage with $j_{i}$ being the rightmost POI covered by $s_{i}$. Then we have the following lemma, which leads to the correctness of the algorithm:

**Lemma** **4.** *When running against a feasible LBTC instance, Algorithm 1 covers the POIs $\left\{1,\;\cdots ,\;j_{i}\right\}$ without using any sensor in $\left\{s_{i+1},\;\cdots ,\;s_{k}\right\}$.*

**Proof.**  We shall prove this claim by induction. When i=1, the lemma is obviously true, as we need only $s_{1}$ to cover the POIs $\left\{1,\cdots ,\;j_{1}\right\}$. Suppose the lemma holds for i=h, then it remains only to show the case for i=h+1. By induction, Algorithm 1 covers the POIs $\left\{1,\;\cdots ,\;j_{h}\right\}$ without using any sensor in $\left\{s_{h+1},\;\cdots ,\;s_{k}\right\}$. Then Algorithm 1 can simply cover POIs $\left\{j_{h}+1,\;\cdots ,\;j_{h+1}\right\}$ by using sensor $s_{h+1}$. Combining with the induction, we covers $\left\{1,\;\cdots ,\;j_{h+1}\right\}$ without using any sensor in $\left\{s_{h+2},\;\cdots ,\;s_{k}\right\}$. This completes the proof. □

We can now prove the following theorem and have the correctness of Algorithm 1:

**Theorem** **2.** *Algorithm 1 returns “feasible” iff the POIs can be completely covered by the sensors within relocation distance D.*

**Proof.**  Suppose Algorithm 1 returns “feasible”, then obviously the produced solution $\left\{x_{i}^{\prime }|i\in \Gamma \right\}$ is truly a coverage, because in the solution the movement of each sensor is bounded by *D* and all the POIs are covered by at least one sensor.

Conversely, suppose there is a coverage for the instance. Then by Lemma 3, there must exist an *s*-ordered coverage, say $\Gamma^{\prime }=\left\{s_{1},\;\cdots ,\;s_{k}\right\}$ which is the set of sensors used to compose the coverage. Following Lemma 4, Algorithm 1 covers POIs $\left\{1,\;\cdots ,\;j_{i}\right\}$ without using any sensor in $\left\{s_{i+1},\;\cdots ,\;s_{k}\right\}$ for every i∈[1,k]. So the algorithm can always find sensors for further coverage, and in the worst case use $s_{i+1}$ to cover the POIs $\left\{j_{i}+1,\;\cdots ,\;j_{i+1}\right\}$. Therefore, the algorithm will eventually find a feasible coverage. This completes the proof. □

## 4. The Complete Algorithms

In this section, we will show how to employ Algorithm 1 to really compute $D^{*}$ the minimum movement bound for LBTC. Firstly, when only considering integer $D^{*}$, we can find it simply by employing the binary search method against a large range that contains $D^{*}$; Secondly, for real number $D^{*}$, we construct a set of size $O(n^{2})$ which arguably contains $D^{*}$, and then eventually finds $D^{*}$ in the set again by the binary search method.

### 4.1. A Simple Binary Search Based Algorithm

The algorithm is simply applying the binary search method to find $D^{*}$ within the range of $\left[1,\;d_{max}\right]$, where $d_{max}$ is the maximum distance between the POIs and the sensors. The main observation is as the following proposition whose correctness is easy to prove:

**Proposition** **3.** If LBTC is feasible, then $D^{*}\leq d_{max}$ holds.

The detailed algorithm is as in Algorithm 2.

**Algorithm 2** The whole algorithm for optimal LBTC.**Input:** A movement bound $D\in Z^{+}$, a set of sensors Γ={1,⋯,n} with positions $\left\{\left(x_{i},y_{i}\right)|i\in {\left[n\right]}^{+}\right\}$,       the sensing radii *r*, a set of POIs P={1,⋯,m} with positions $p_{1}⪯p_{2}⪯\cdots ⪯p_{m}$, where $p_{j}$ is       the position for j∈P;**Output:** The minimized maximum movement of the sensors together with new positions $\left\{x_{i}^{\prime }|i\in {\left[n\right]}^{+}\right\}$ 1: Set $ub:=d_{max}$ and lb:=1, where $d_{max}$ is the maximum distance between the sensors and the POIs; 2: **If** there exists no coverage returned from calling Algorithm 1 wrt **$D=d_{max}$ then** 3:     Return “infeasible”; 4: **EndIf** 5: Set $tmp:=\left\lceil \frac{lb+ub}{2}\right\rceil$; 6: **While**
ub−lb>1
**do** 7:     **If** there exists no coverage returned from calling Algorithm 1 wrt **D=tmp then** 8:        Set lb:=tmp and $tmp:=\left\lceil \frac{lb+ub}{2}\right\rceil$; 9:     **Else**10:        Set ub:=tmp and then $tmp:=\left\lceil \frac{lb+ub}{2}\right\rceil$;11:     **EndIf**12: **End While**13: Return the result of call Algorithm 1 wrt **D=tmp**.


For the correctness and time complexity of Algorithm 2, we immediately have the following lemma:

**Lemma** **5.** Using binary search and employing Algorithm 1 for $O(\log d_{max})$ times, Algorithm 2 will compute the optimum movement $D^{*}$ within time complexity $O(n\log n\log d_{max})$.

### 4.2. An Improved Algorithm via Discrete Binary Search

**Figure 2:**
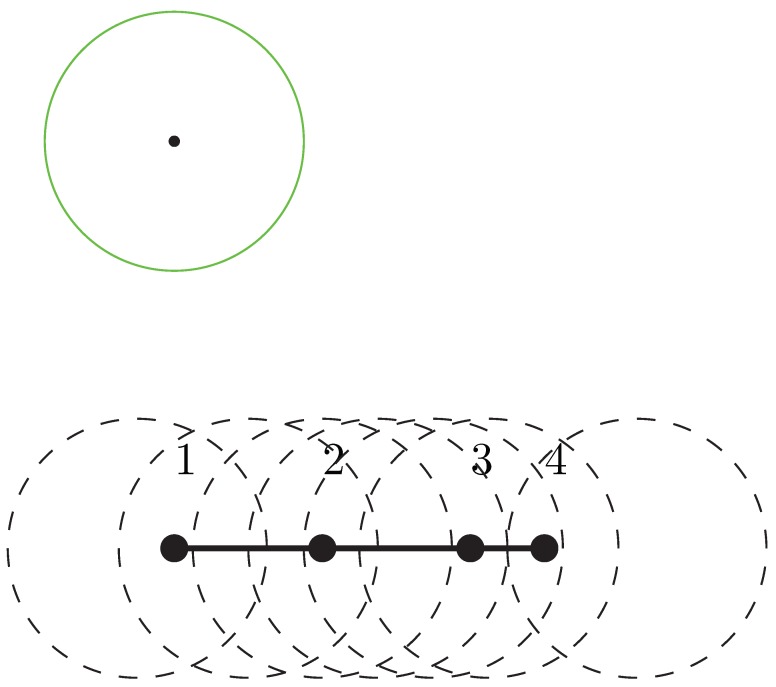
An example of combinations: For POIs on the line segment and the radius of sensors (in green) as depicted, the collection of combinations is Φ={{1},{1,2},{2},{2,3},{2,3,4},{3,4},{4}}.

In this subsection, we shall show the time complexity of our algorithm can be further improved via a more sophisticated implementation over the binary search. The key observation is that, we need only to apply a binary search over a set of discrete values which arguably contain the optimum min-max movement $D^{*}$. Let $\Phi =\{c_{1},\;\cdots ,\;c_{t}\}$ be the set of possible combinations, that is, all possible different combinations covered by a sensor (An example of combinations is as depicted in Figure 2). Let $d_{ij}$ be the minimum movement using sensor *i* to cover combination $c_{j}$. Then we have the following lemma:

**Lemma** **6.** *Let $d_{opt}$ be an optimal solution to the uniform 2D-LBTC problem. Then $d_{opt}\in \Psi =\left\{d_{ij}|i\in \Gamma ,\;c_{j}\in \Phi \right\}$.*

**Proof.**  Suppose the lemma is not true. Then let $d_{max}=\max_{d}\left\{d|d\in \Psi ,\;d<d_{opt}\right\}$. First we show that under maximum distance $d_{max}$ and $d_{opt}$, every sensor *i* covers an identical collection of combinations. That is because every POI, which sensor *i* can cover under movement bound $d_{opt}$, can also be covered by sensor *i* under movement bound $d_{max}$ ( as $d_{ij}\leq d_{max}$ iff $d_{ij}<d_{opt}$), and conversely every POI, which cannot be covered by sensor *i* under $d_{max}$, can not be covered by the same sensor within the movement bound $d_{opt}$ ($d_{ij}>d_{max}$ iff $d_{ij}>d_{opt}$). Therefore, a feasible coverage solution under maximum movement $d_{opt}$ would also remain feasible under $d_{max}$. This together with $d_{max}<d_{opt}$ contradicts with the fact that $d_{opt}$ is an optimal solution to the problem. □

Following the above lemma, our algorithm will first compute $⊕=\{c_{1},\;\cdots ,\;c_{t}\}$ the set of possible combinations; Then compute all the distances between each sensor in Γ and each combination in C, which is $\Psi =\{d_{ij}|i\in \Gamma ,\;c_{j}\in \Phi \}$; Later sort the distance in Ψ in non-decreasing order and apply the binary search method to Ψ by using Algorithm 1 as a subroutine. Eventually, the algorithm finds a minimum $d_{ij}^{*}\in \Psi$, such that by setting the bound the maximum movement $D=d_{ij}^{*}$ there exists a relocation of the sensors to cover all the POIs.

According to the main structure of the complete algorithm above, we shall first compute ⊕. The key idea of the computation is to find all the pairs of POIs which can be the first and the final POIs covered by a sensor. The most important part of the computation is to exclude those pairs of POIs, say *i* and *j*, for which i−1 and j+1 are too close, i.e., d(i−1,j+1)≤2r. In the case, to cover *i* and *j*, either i−1 or j+1 will be also covered, and hence *i* and *j* can not respectively be the first and the final POIs covered by a sensor. The detailed algorithm for computing Φ and the complete algorithm are formally stated in Algorithms 3 and 4.

**Algorithm 3** A fast algorithm for constructing combinations.**Input:** A set of POIs P={1,⋯,m} on the line segment with positions $p_{1}⪯p_{2}⪯\cdots ⪯p_{m}$,       where $\left(p_{j},\;0\right)$ is the position for j∈P, and a real number *r* that is the radii of the sensors;**Output:** The set of combinations $\Phi :=\{c_{1},\;\cdots ,\;c_{t}\}$. 1: Set Φ:=∅, $p_{0}:=-2r$ and $p_{m+1}=p_{m}+2r$; 2: **For** POI j=1 to |P|
**do** 3:    **For**
i:=j to |P|
**do** 4:        **If $p_{i}-p_{j}\leq 2r$** and $p_{i+1}-p_{j-1}\geq 2r$
**then** 5:            Φ:=Φ∪{{j,⋯,i}}; 6:       **EndIf** 7:    **EndFor** 8: **EndFor** 9: Return Φ and terminate.


**Algorithm 4** A fast algorithm for LBTC.**Input:** A set of sensors Γ={1,⋯,n} with original positions $\left\{\left(x_{i},y_{i}\right)|i\in {\left[n\right]}^{+}\right\}$ and an identical       sensing radii *r*, a set of POIs P={1,⋯,m} with positions $p_{1}⪯p_{2}⪯\cdots ⪯p_{m}$ on the line       segment, where $p_{j}$ is the position for j∈P;**Output:** A minimum movement *D* under which the sensors can be relocated to covered all POIs in *P*. 1: Set Ψ:=∅ and compute $\Phi :=\{c_{1},\;\cdots ,\;c_{t}\}$ the collection of combinations by Algorithm 3; 2: **For** each sensor *i*
**do** 3:    **For** each combination $c_{j}\in \Phi$
**do** 4:       Compute $d_{ij}$, the minimum movement needed when using sensor *i* to cover $c_{j}$; 5:       Set $\Psi :=\Psi \cup \{d_{ij}\}$; 6:    **EndFor** 7: **EndFor** 8: Sort Ψ in a non-decreasing order and set lb:=1 and ub:=|Ψ|, to prepare for binary search. 9: Use Ψ[1] as the movement bound (i.e., *D*) and call Algorithm 1;      /*Note that Ψ[1] is the smallest element in Ψ. */10: **If** there exists a feasible coverage under movement bound Ψ[1]
**then**11:    Return Ψ[1] as the optimum movement bound;12: **End if**13: **While ub−lb>1 do**14:    Set **$idx:=\left\lceil \frac{lb+ub}{2}\right\rceil$**;15:    Use Ψ[idx] as the movement bound (i.e., *D*) and call Algorithm 1;        /*Ψ[idx] is the idxth smallest element in Ψ. */16:    **If** there exists a feasible coverage under movement bound Ψ[idx]
**then**17:       Set ub:=idx;18:    **Else**19:       Set lb:=idx;20:    **End if**21: **Endwhile**22: Return Ψ[idx] as the optimum movement bound.

**Lemma** **7.** *Algorithm 3 returns*Φ with *|Φ|=O(m).*

**Proof.**  W.l.o.g. assume the combinations in Φ are sorted, such that for each $c_{i}$ and $c_{i+1}\in \Phi$, $l\left(c_{i}\right)\leq l\left(c_{i+1}\right)$ and $g\left(c_{i}\right)\leq g\left(c_{i+1}\right)$, where $l(c_{i})$ and $g(c_{i})$ are respectively the leftmost and rightmost POIs in $c_{i}$. Then, following the construction of the combinations as in Algorithm 3, if a POI *j* appears in $c_{i}$ and $c_{i+\Delta }$, then for any k∈[i,i+Δ], $j\in c_{k}$. That is, a POI can result in at most two different combinations, one when the POI is added and the other when the POI is removed. Therefore, there are at most 2m combinations in Φ. □

**Lemma** **8.** *The time complexity of Algorithm 4 is O(mn(logm+logn)).*

**Proof.**  From Lemma 7, |Φ|=O(m) holds, so we apparently have |Ψ|=O(mn). Then, it takes O(|Ψ|log|Ψ|)=O(mnlogmn)=O(mn(logm+logn)) time to sort the elements in Ψ, provided merge sort is used [15]. Besides, the while-loop from Step 12 to Step 20 will be repeated for at most O(logm+logn) times, each of which takes O(nlogn) time to run Algorithm 1. Therefore, the total time complexity of the algorithm is O(mn(logm+logn)). □

Following Lemma 6, we immediately have the correctness of Algorithm 4 as below:

**Theorem** **3.** *Algorithm 4 produces an optimum solution to the LBTC problem.*

## 5. Experiments

In this section, we shall numerically evaluate the practical performance of both Algorithm 2 (the simple Algorithm based on Binary Search, denoted as ABS) and Algorithm 4 (the Algorithm employing Binary Search over a specially constructed Discrete set, denoted as ABSD) by comparing its runtime and practical solution quality with an optimal algorithm baseline—an exact algorithm by solving an Integer Linear Programming (ILP) formulation which produces optimal solution for LBTC. The experiments are carried out on a platform with Intel I5 CPU and 8G DDR3 Memory, using Windows 10 Operating system, and the algorithms are implemented in C++ (The code is available upon request) adopting the *CPLEX* library (https://www.ibm.com/analytics/cplex-optimizer) to solve the ILP.

### 5.1. An ILP Formulation for LBTC

Let variable $x_{ij}$ represents whether combination *j* is covered by sensor *i* and *D* be the maximum movement. Then we have the ILP formulation for LBTC as below (Denoted as ILP (1)):

$$\begin{array}{cc} \min & D \end{array}$$

(2) $$\begin{array}{ccc} s.t. & \sum_{i\in \Gamma }x_{ij}\geq 1 & \forall j\in \Phi \end{array}$$

(3) $$\begin{array}{cc} \sum_{j\in \Phi }x_{ij}\leq 1 & \forall i\in \Gamma \end{array}$$

(4) $$\begin{array}{cc} \sum_{j\in \Phi }x_{ij}d_{ij}\leq D & \forall i\in \Gamma \end{array}$$

(5) $$\begin{array}{cc} x_{ij}\in \left\{0,\;1\right\} & \forall i\in \Gamma ,\;\forall j\in \Phi \end{array}$$

In the above ILP formula, Inequality (2) guarantees that each POI is covered; Inequality (3) ensures that each sensor is used for at most once; Inequality (4) means *D* is the maximum movement. It is then easy to obtain the following property:

**Lemma** **9.** *An optimal solution to ILP (1) is an optimum solution to LBTC, and vice versa.*

### 5.2. Numerical Experiments

In the numerical experiments, we shall first evaluate the runtime of the algorithms with *n* sensors distributed in the plane, where the radius of the sensors are set 10, the number of sensors *n* is set in a range [100,900], and the positions of the sensors are uniformly and randomly distributed in a rectangle along the line segment of a length *L* that is set proportional to the number of the sensors. The POIs are randomly and uniformly distributed on the line segments, and are with a number proportional to the number of the sensors.

**Figure 3:**
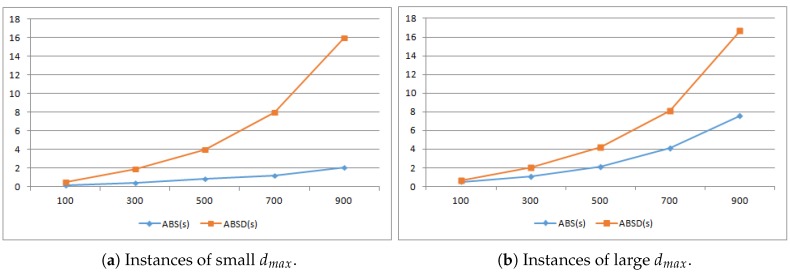
Runtime comparison between ABS and ABSD.

**Figure 4:**
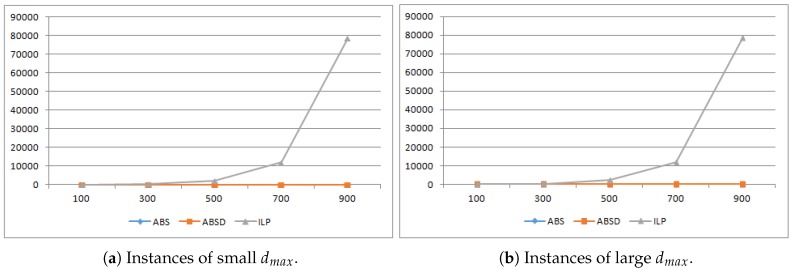
Runtime comparison of ABS and ABSD against ILP, where ABS and ABSD overlap as one line because they are very close to each other comparing to ILP.

In both Figure 3 and Figure 4, we compare the runtime of ABS, ABSD and ILP in seconds. For each n∈{100,300,500,700,900}, to obtain the runtime, we produced 1000 different instances of LBTC, against which we respectively ran the three algorithms and used the average runtime of the 1000 runs as the final runtime. Note that as we shall choose L=n∗r/4, the generated LBTC instances are all feasible, i.e., in every instance, all the POIs can be completely covered.

In Figure 3, the experiments compare the runtimes of ABS and ABSD for different $d_{max}$. When $d_{max}$ is small, i.e., when the rectangle where the sensors distribute is 60∗L along the line segment, the runtime of ABS is much better than ABSD (i.e., about 8 times faster as depicted in Figure 3a). However, when $d_{max}$ is large (i.e., when the rectangle is 10,000 ×5L), the runtime of ABSD then is very close to ABS that about 1.9 times of that of ABS as depicted in Figure 3b. This result fits the time complexity $O(n\log n\log d_{max})$ and O(mn(logm+logn)), where *m*, *n* and $d_{max}$ are respectively the numbers of the POIs, sensors and the maximal distance between the POIs and the sensors. In addition, the runtime of ABS depends on $d_{max}$ while ABSD is insensitive to the distance between the sensors and the POIs, which again coincides with the theoretical runtime analysis of ABSD. When $d_{max}$ grows, the runtime gap between ABS and ABSD shrinks.

As depicted in Figure 4, when the problem size grows, the runtime of ABS and ABSD both grows slow comparing to ILP whose runtime grows fast that it becomes nearly not applicable when there are more than 500 sensors (its runtime is more than thousands of seconds (The runtime here is the average of only several runs instead of 1000 runs, because it is too large.) as depicted both in Figure 4a,b). Notably, the runtime of ABS and ABSD are both under 5 s at 500. So both ABS, ABSD have a significant runtime advantage comparing to the ILP baseline. Also, the runtime of ILP is insensitive to the distance between sensors and POIs, as the runtime are almost the same for both instances of small and large $d_{max}$.

**Figure 5:**
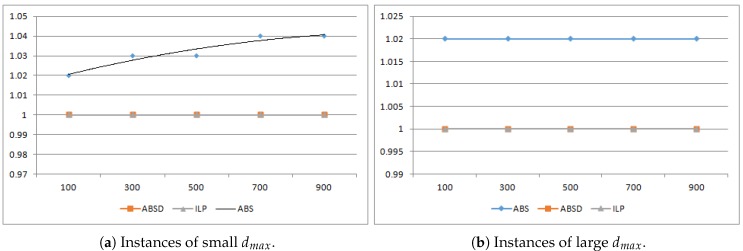
Comparison of practical solution quality of the algorithms.

Note that, although the ABS has the runtime among all the three algorithms, it can only produce optimal solutions when the movement is an integer. However, in the experiments, the distance are real numbers, so ABS only produce approximation solutions. On the other hand, according to our theoretical analysis, ABSD always produces optimal solutions. As depicted in Figure 5a,b, throughout all the thousands of LBTC instances, every solution produced by ABSD has a maximum movement coincides with the optimal solution produced by ILP. In contrast, ABS produces only approximation solutions. When for the instance of small $d_{max}$, the solutions produced by ABS are with an average maximum movement about 1.04 times of the optimal solutions, as illustrated in Figure 5a; while $d_{max}$ grows large, the approximation ratio becomes even better that it is 1.02, as illustrated in Figure 5b.

## 6. Conclusions

In this paper, we first proved that 1D-LBTC is NP-hard when the radius of the sensors are not identical. It is worth to note that this result is interesting because 1D-LBC problem can be efficiently solved in a polynomial time. Then, we designed an algorithm for decision LBTC with uniform radius, and consequently proposed an algorithm for really solving LBTC based on the binary search method. Moreover, we improved the binary search method to a runtime $O(n^{2}\log n)$ by observing that the optimum movement bound is within the set of distances between the POIs and the sensors. We also evaluated the practical performance of our algorithms via numerical experiments. We are currently investigating how to further improve the runtime of the algorithm.

## Figures and Tables

**Table 1:** Notations used in the paper.

Notations	Description
LBTC	Brief for the min-max 2D Line Boundary Target Coverage problem
Decision LBTC	The problem that only determines whether LBTC is feasible under given *D*
LBC	Brief for the min-max 2D Line Boundary Coverage Problem
1D-LBTC	LBTC but with the original positions of the sensors on the line
1D-LBTC	LBC but with the original positions of the sensors on the line
POI	Points Of Interest which are the targets to be covered
3-partition	A combinatorial optimization problem that is know strongly NP-complete
*L*	The length of the line segment where the POIs are placed
*D*	The bound of the maximum movement of the sensors
$D^{*}$	The minimum one among all feasible *D*
*P*	The set of POIs
$p_{j}$	The position of j∈P on the line segment
Γ	The set of sensors
$d_{max}$	The maximum distance between the POIs and the sensors
$d_{ij}$	The distance between POI *j* and sensor *i*
$l_{i}$	The leftmost point sensor *i* can cover under the given movement bound *D*
$g_{i}$	The rightmost point sensor *i* can cover under the given movement nbound *D*
l(C)	The leftmost POI of the POI set *C*
g(C)	The rightmost POI of the POI set *C*
Φ	The possible maximal sets of POIs (called combinations) that can be covered by a single sensor
Ψ	$\Psi =\{d_{ij}|i\in \Gamma ,\;c_{j}\in \Phi \}$ is the set of distances between the combinations and the sensors
